# External Carotid Artery Pseudoaneurysm: A Rare Cause of Ischemic Stroke via Internal Carotid Artery Compression—A Case Report

**DOI:** 10.1155/crnm/4572560

**Published:** 2026-09-11

**Authors:** Harsimran Kaur, Nihas Mateti, Gabrielle Feher, Romil Singh, Russell Cerejo

**Affiliations:** ^1^ Department of Neurology, University of Pittsburgh Medical Center, Pittsburgh, Pennsylvania, USA, upmc.com; ^2^ Department of Neurology, Allegheny Health Network, Pittsburgh, Pennsylvania, USA, ahn.org; ^3^ Department of Medicine, Drexel Medical School, Philadelphia, Pennsylvania, USA, duhs.edu.pk

## Abstract

Pseudoaneurysms (PSAs) typically result from trauma, but their complications beyond rupture and bleeding remain understudied. We present a case of a 75‐year‐old male with atrial fibrillation who sustained a gunshot wound to the face, resulting in a left internal maxillary artery PSA. The patient was initially managed conservatively but developed right‐sided hemiplegia and aphasia 14 days postinjury. CT angiography revealed near‐complete occlusion of the left cervical internal carotid artery (ICA) due to extrinsic compression from the expanding PSA, originating from a branch of the external carotid artery (ECA). Emergent digital subtraction angiography confirmed ICA occlusion, and successful embolization of the PSA resulted in recanalization of the ICA and immediate neurological improvement. This case highlights a rare but significant complication of ECA‐PSAs: ICA compression leading to ischemic stroke. This report underscores the importance of considering PSA‐induced ICA compression as a potential cause of ischemic stroke in trauma patients, especially when neurological deficits develop. Prompt diagnosis and intervention are crucial for optimal patient outcomes.

## 1. Introduction

A pseudoaneurysm (PSA), or false aneurysm, is characterized by a rupture in the continuity of the arterial wall, forming an aneurysmal sac [1]. Extracranial carotid artery PSA (ECA‐PSA) is an uncommon type of peripheral arterial aneurysm and comprises less than 1% of all arterial aneurysms [2]. It is estimated that > 90% of ECA aneurysms are PSAs rather than true aneurysms [3]. The most common causes of ECA‐PSA include penetrating or acute blunt neck trauma, which can lead to life‐threatening complications, such as rupture, massive hemorrhage, or airway compromise [4]. However, an underrecognized yet significant complication is the extrinsic compression of adjacent vascular structures, which can result in ischemic stroke [5].

Stroke is typically associated with embolic or thrombotic mechanisms, but mechanical compression of the internal carotid artery (ICA) by ECA‐PSA remains an unusual and underreported etiology. Diagnosing such cases can be challenging, often requiring advanced vascular imaging to identify extrinsic compression. In this case report, we present a rare instance of ischemic stroke secondary to ICA compression caused by an ECA‐PSA.

## 2. Case Description

A 75‐year‐old male with a history of atrial fibrillation (managed with apixaban), chronic kidney disease, and hypertension presented to the emergency department following a penetrating gunshot wound to the left face. The injury resulted in extensive trauma involving the left masticator and mandibular spaces.

**FIGURE 1:**
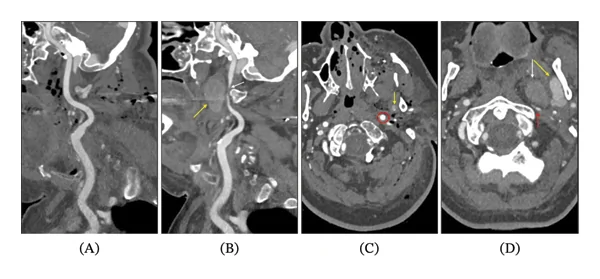
A CT angiogram of 75‐year‐old patient who presented to the hospital for a gunshot wound to the left side of the face. Maximum intensity projection reconstruction of the left internal carotid artery at Day 0 (A) and Day 14 (B) shows a short segment high‐grade or near‐complete occlusion of the distal cervical left internal carotid artery (white arrow), with distal reconstitution and normal vessel caliber. The yellow arrow points to pseudoaneurysm of the internal mammillary artery branch of the external carotid artery. (C) Axial CT angiogram of the patient’s neck at Day 0. The yellow arrow shows wispy contrast enhancement in the proximal portion of the left internal maxillary artery suggestive of a small pseudoaneurysm or subtle extravasation. The red circle shows widely patent internal carotid artery. (D) Axial CT angiogram of the patient’s neck at Day 14. The red arrow shows a short segment high‐grade or near‐complete occlusion of the distal cervical left internal carotid artery. The yellow arrow demonstrates arterial hyperenhancement, and the white arrow shows venous phase enhancement concerning for venous and arterial pseudoaneurysms from penetrating injury.

The initial CT angiogram of the head and neck revealed wispy contrast enhancement in the proximal portion of the left internal maxillary artery. This was suggestive of a small PSA or subtle extravasation, which caused mild extrinsic compression of the distal left ICA (Figure 1A–C). Since the patient was asymptomatic from the extrinsic compression, he was treated conservatively with the continuation of apixaban and follow‐up imaging planned in 2‐3 weeks.

**FIGURE 2:**
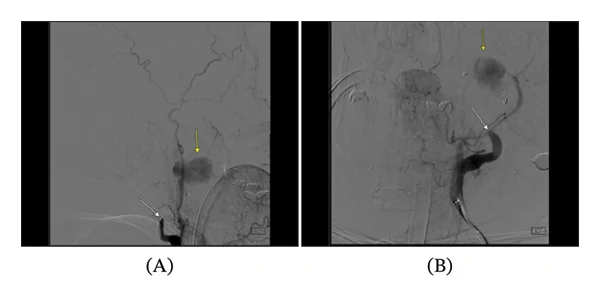
Digital subtraction angiography of the left common carotid artery in a 75‐year‐old trauma patient with an internal maxillary artery pseudoaneurysm (Panel A shows lateral view and Panel B shows coronal view). The white arrows indicate occlusion of the proximal left cervical internal carotid artery. The yellow arrows show the compression from left internal maxillary artery pseudoaneurysm/contained rupture.

On hospital Day 14, while undergoing hemodialysis, the patient experienced an acute onset of right‐sided weakness and aphasia. A neurological examination revealed a National Institutes of Health Stroke Scale (NIHSS) score of 17. A repeat CT angiography demonstrated high‐grade, near‐complete occlusion of the left cervical ICA, likely secondary to extrinsic compression from an enlarging PSA located in the left parapharyngeal space (Figures 1B–D and 2A).

The patient underwent emergent digital subtraction angiography (DSA). Diagnostic angiography confirmed the left cervical ICA occlusion was likely due to external compression from the left internal maxillary artery PSA. Crucially, there was no angiographic evidence of intrinsic mural injury, such as an intimal flap, double lumen, or string sign, excluding ICA dissection as a primary or contributing mechanism. Because the occlusion was dynamic, purely compressive, and nonthrombotic, mechanical thrombectomy or thrombolysis was not indicated. Furthermore, primary carotid stenting was deferred; deploying a stent in a healthy, compressed artery would carry unnecessary risks of in‐stent thrombosis and require dual antiplatelet therapy, which was undesirable in the setting of recent penetrating trauma and apixaban use.

Instead, isolated embolization of the maxillary PSA was pursued to relieve the mass effect. The intervention utilized a coaxial approach. An intermediate catheter (058 Navien) was positioned in the midleft ECA to provide proximal flow control, while a microcatheter (SL 10) was advanced directly into the left proximal internal maxillary artery. The microcatheter was prepped with 5% dextrose in water (D5 W), and the PSA was embolized using a 1:1 mixture of TRUFILL n‐butyl cyanoacrylate (n‐BCA) and Lipiodol. Once a satisfactory cast filled the PSA, gentle aspiration was applied to snap the glue column, and the microcatheter was removed.

**FIGURE 3:**
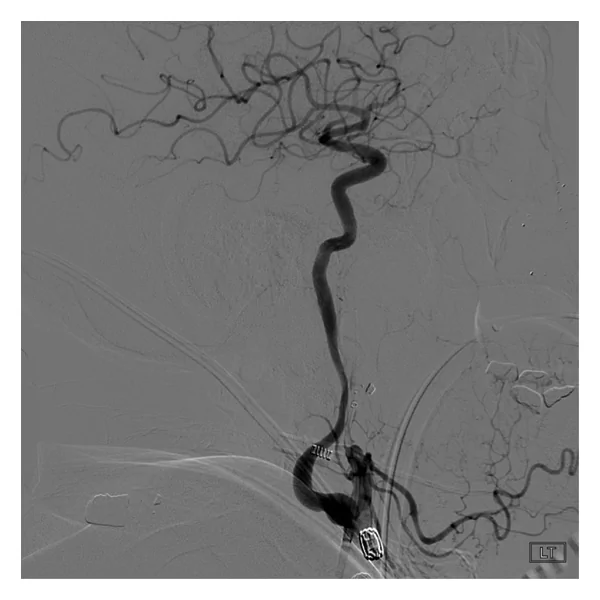
Digital subtraction angiography of left internal carotid artery in a 75‐year‐old trauma patient with an internal maxillary artery pseudoaneurysm following successful n‐butyl cyanoacrylate embolization. Embolization of the left internal maxillary artery restored flow in the left cervical internal carotid artery with no identified intracranial branch occlusion. There is still mild nonflow limiting narrowing in the midcervical internal carotid artery at the previous site of occlusion likely from the pseudoaneurysm.

Postintervention DSA demonstrated complete resolution of the extrinsic mass effect and immediate restoration of normal antegrade ICA flow (mTICI 3 complete perfusion), with only mild residual narrowing at the site of the previous compression (Figure 3, annotated with arrows highlighting the compression‐recanalization sequence) (Figure 4 Video 1). There was no evidence of intracranial branch occlusion or capillary wedge defects to suggest distal microembolization or n‐BCA reflux.

**FIGURE 4:**
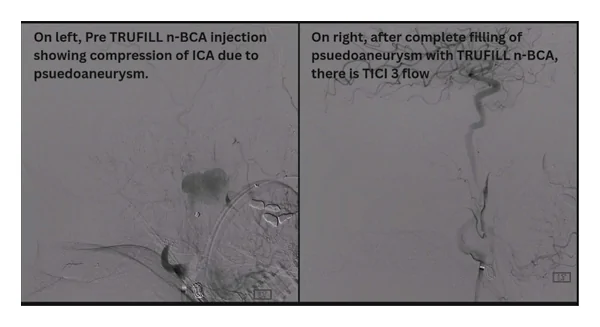
The full video can be accessed in the Supporting Information. Dynamic digital subtraction angiography (DSA) demonstrating the compression‐recanalization sequence. (Left) Preintervention DSA of the left common carotid artery reveals severe flow limitation in the proximal left cervical internal carotid artery (ICA) secondary to dynamic extrinsic compression from an enlarging left internal maxillary artery pseudoaneurysm. (Right) Postintervention DSA following targeted embolization of the pseudoaneurysm sac with TRUFILL n‐butyl cyanoacrylate (n‐BCA). Following the successful n‐BCA cast and relief of the mass effect, there is immediate restoration of widely patent antegrade flow in the left cervical ICA, achieving a modified treatment in cerebral ischemia (mTICI) Grade 3 perfusion.

Immediately after embolization, the patient’s neurological status improved, and he demonstrated the ability to lift his right arm and leg against gravity (NIHSS improved from 17 to 7). A repeat noncontrast CT of the head did not show acute cerebral infarction changes in the left ICA territory. However, it did reveal scattered subarachnoid hemorrhage (SAH) in the left frontal lobe. Given the definitive absence of distal emboli on DSA, this small, asymptomatic SAH was presumed secondary to hemodynamic reperfusion injury following the rapid normalization of flow to a hypoperfused left hemisphere. The patient’s apixaban was discontinued, and the SAH resolved over the next few days with conservative management. The patient was initiated on low molecular weight heparin on postoperative Day 7 after a stable CT head was confirmed and was then transitioned to apixaban following 48 h of continued stability.

A brain MRI would have helped precisely identify the extent of any ischemic core but was contraindicated due to retained bullet fragments. During the remainder of his hospitalization, the patient experienced no recurrent ischemic or hemorrhagic events. He was discharged to an inpatient rehabilitation facility with a discharge mRS of 4. Unfortunately, his rehabilitation course was complicated by multiple medical comorbidities, including end‐stage renal disease and aspiration pneumonia, and he passed away prior to his 90‐day mRS assessment.

## 3. Discussion

Although ischemic stroke is most associated with thromboembolic events, this case highlights a rare mechanism—direct mechanical compression of the ICA by an ECA‐PSA, resulting in ischemic stroke. The PSA developed following a gunshot wound to the left face and caused extensive soft tissue damage in the masticator and mandibular spaces. As the PSA expanded, it compressed the adjacent ICA, leading to severe stenosis and impaired cerebral perfusion.

Carotid artery PSAs arise most frequently from penetrating or blunt trauma (e.g., motor vehicle accidents) but also from neck compression, hyperextension/rotation injuries, skull base trauma, or iatrogenic causes [6]. Kraus et al. [7] reported a 0.27% incidence of carotid injury in trauma patients, with PSAs occurring in approximately 25% of these cases. Analysis of 141 ECAs demonstrated a predilection for the ICA, with ECA involvement being rare (seen in only one patient) [8]. The common symptoms described in the cohort were painless mass, transient ischemic attacks, and rupture [8]. The etiology of transient ischemic attack or stroke is thought to be embolization. PSA can also cause compressive symptoms such as dysphagia and cranial nerve palsies [9]. Cox et al. [10] evaluated 124 penetrating head and neck injuries and found 10 ECA‐PSAs with hemorrhagic complications but no ischemic strokes from ICA compression.

Our case represents a rare etiology of ECA‐PSA causing mechanical ICA compression and a subsequent stroke. Previously, there are reports of compression from other sources causing vascular compromise and stroke [11–13]. For example, mechanical compression of the ICA can occur in patients with an anatomical variation known as a “wandering carotid artery” [14]. This variation, characterized by aberrant ICA positioning within the neck, can cause life‐threatening complications, including hemorrhage during surgical procedures (e.g., tonsillectomy and adenoidectomy) [14]. While spontaneous resolution of ICA compression has been reported [11], there is a documented case of recurrent ischemic stroke secondary to dynamic compression by the hyoid bone and thyroid cartilage that required surgical intervention [13]. In the present case, the patient’s symptoms were resolved after using surgical interventions (embolization) to treat the ECA‐PSA and consequent ICA decompression, similar to how other cases of compression‐induced vascular compromise were treated [12].

Early recognition of PSA‐induced stroke requires advanced vascular imaging. In this case, CT angiography and DSA were critical in identifying the PSA and its compressive effects on the ICA. While Doppler ultrasound can distinguish vascular lesions from solid masses, its utility in trauma is often limited by deep anatomical location, swelling, and retained foreign bodies [15]. This reinforces the importance of CT angiography and DSA as first‐line imaging tools for vascular trauma.

Currently, no randomized controlled trials compare open surgical versus endovascular repair as a treatment for ECA‐PSAs. The endovascular approach is favored for traumatic PSAs due to reduced risk associated with open surgery in the setting of tissue distortion, edema, and infection [10]. Successful coil embolization has been reported in multiple case series [16, 17]. Wang et al. [17] further demonstrated the efficacy of endovascular techniques, including coil embolization and the use of Onyx or polyvinyl alcohol particles, for various locations of traumatic ECA and branch PSAs. This approach offers advantages, such as simultaneous diagnosis and treatment, the ability to manage multiple PSAs, and a shorter procedure duration with minimal scarring [17]. Surgical ligation also remains an option, particularly for traumatic scalp PSAs [17]. The choice of embolic agent (e.g., n‐butyl cyanoacrylate and Onyx) may vary depending on the specific clinical scenario. In our case, the patient was treated with n‐butyl cyanoacrylate embolization, which successfully restored ICA blood flow and improved the patient’s neurological status.

From a therapeutic standpoint, endovascular embolization provided a rapid, minimally invasive solution, restoring ICA patency while avoiding the risks of open surgery. Future studies should examine the prevalence of PSA‐related ICA compression, compare embolization versus surgical repair outcomes, and evaluate whether early vascular imaging in high‐risk trauma patients could reduce stroke incidence.

This case reinforces the need for increased clinical suspicion of PSA‐induced vascular compression in trauma patients with stroke‐like symptoms. Incorporating early vascular imaging into trauma protocols could improve outcomes by ensuring timely diagnosis and intervention, ultimately preventing devastating ischemic complications.

## Funding

This study was funded by Allegheny Health Network’s Health System Publication Support Office (HSPSO) provided assistance with editing and formatting the manuscript. The HSPSO was funded by Highmark Health (Pittsburgh, PA, United States of America).

## Consent

Patient consent was obtained for this publication.

## Conflicts of Interest

The authors declare no conflicts of interest.

## Supporting Information

Additional supporting information can be found online in the Supporting Information section.

## Supporting information


**Supporting Information** Video 1: Dynamic digital subtraction angiography (DSA) demonstrating the compression‐recanalization sequence. (Left) Preintervention DSA of the left common carotid artery reveals severe flow limitation in the proximal left cervical internal carotid artery (ICA) secondary to dynamic extrinsic compression from an enlarging left internal maxillary artery pseudoaneurysm. (Right) Postintervention DSA following targeted embolization of the pseudoaneurysm sac with TRUFILL n‐butyl cyanoacrylate (n‐BCA). Following the successful n‐BCA cast and relief of the mass effect, there is immediate restoration of widely patent antegrade flow in the left cervical ICA, achieving a modified Treatment in Cerebral Ischemia (mTICI) Grade 3 reperfusion.

## Data Availability

Data sharing is not applicable to this article as no new data were created or analyzed in this study.
